# The Response of Salinity Stress-Induced *A. tricolor* to Growth, Anatomy, Physiology, Non-Enzymatic and Enzymatic Antioxidants

**DOI:** 10.3389/fpls.2020.559876

**Published:** 2020-10-16

**Authors:** Umakanta Sarker, Shinya Oba

**Affiliations:** ^1^Department of Genetics and Plant Breeding, Faculty of Agriculture, Bangabandhu Sheikh Mujibur Rahman Agricultural University, Gazipur, Bangladesh; ^2^Laboratory of Field Science, Faculty of Applied Biological Sciences, Gifu University, Gifu, Japan

**Keywords:** ascorbate and ascorbate redox, catalase, ascorbate peroxidase, guaiacol peroxidase, superoxide dismutase, antioxidant activity, phenolics, carotenoid

## Abstract

An investigation was carried out to elucidate growth, anatomical, physiological, and major ROS detoxification pathways involved in the tolerance of *A. tricolor* under salinity stress. Both VA14 and VA3 varieties exhibited the reduction in relative water content (RWC), photosynthetic pigments, growth, increased electrolyte leakage (EL), and leaf anatomy adaptation under salinity stress, whereas VA14 was well adapted and performed better compared to VA3. Higher ROS accumulation was demonstrated in the sensitive variety (VA3) in comparison to the tolerant variety (VA14). Salinity stress changed the cellular antioxidant pool by increasing total carotenoids, ascorbate, proline, total polyphenol content (TPC), total flavonoid content (TFC), and total antioxidant capacity (TAC) in both varieties. Although a higher increment was demonstrated in the tolerant variety, the proline increment was much more pronounced in the sensitive variety. Non-enzymatic antioxidant, ascorbate, carotenoids, TPC, TFC, TAC, and antioxidant enzymes SOD and APX were noted to be a major H_2_O_2_ detoxifier in the tolerant *A. tricolor* variety, where there is a comparatively lower H_2_O_2_ load. It was complemented by GPOX and CAT activity at a comparatively higher H_2_O_2_ load (in the sensitive variety). SOD contributed to the dismutation of superoxide radical (SOR) both in the tolerant and sensitive varieties; however, it greatly contributed to the dismutation of SOR in the tolerant variety. The increase in SOD, ascorbate, and APX makes it predominantly evident that SOD and the AsA–GSH cycle had greatly contributed to quench reactive oxygen species (ROS) of the tolerant variety of *A. tricolor*.

## Introduction

Plant productivity and growth are seriously impeded by salinity stress. This is predicted to increase along with global climate change through a rise in sea levels. Approximately, soil salinity affects 7% of total land and 20% of arable land across the globe. If the existing scenario of salt stress continues, by 2050, fifty percent of land under cultivation may be lost through soil salinization. In the meantime, worldwide annual farming production losses from salt-affected lands are over US$12 billion and expanding (Flowers et al., 2010). Therefore, it is of vital need to increase salt tolerance of crops for sustainable agriculture.

In Bangladesh, Southeast Asia, and Africa, leaves of *A. tricolor* L. are popularly used as inexpensive and common vegetables. It is commonly cultivated in the saline prone area in the coastal belt including semiarid and arid regions. It is the cheapest unique source of betalains including flavonoids, vitamins, and phenolics as natural antioxidants. It protects numerous diseases including arthritis, cancer, neurodegenerative disease, atherosclerosis, retinopathy, cardiovascular diseases, cataracts, and emphysema (Repo-Carrasco-Valencia et al., 2010; Steffensen et al., 2011). It demonstrated high adaptability to abiotic stresses (Rana et al., 2007; Sarker and Oba, 2018a; Sarker and Oba, 2018b; Sarker and Oba, 2018c; Sarker and Oba, 2018d; Sarker and Oba, 2018e).

In plants, salt stress induces responses causing biochemical, physiological, morphological, and molecular changes. Salinity hinders the development and growth of plants through osmotic stress, causing specific ion (Na^+^ and Cl^-^) toxicity (Munns et al., 2006), a disturbing major cytosolic enzymes activity, by hampering intracellular potassium homeostasis. In plant cells, salinity stress creates oxidative stress through aggravating the excessive free oxygen radicals (hydroxyl radicals, (OH•); hydrogen peroxide, H_2_O_2_; singlet oxygen, ^1^O_2_; alkoxyl radical, RO; and superoxide radical, (O_2_^•-^) (SOR)) (Hernández et al., 1993; Hernandez et al., 1995) from mitochondrial respiration, photosynthesis, and photorespiration pathway (Sharma et al., 2013). ROS restricts the normal metabolism of cells and destroys through oxidation of DNA, proteins, lipids, and other macromolecules of the cell (Perez-Lopez et al., 2009; Gill and Tuteja, 2010; Wang et al., 2012; Ahanger et al., 2020). Peroxisomes, chloroplasts, endoplasmic reticulum, plasma membranes, and mitochondria are the main source of free oxygen radicals in plant cells (Asada, 1999; Foyer and Noctor, 2009).

Salinity stress results in the accumulation of Na^+^ that participates with K^+^ to bind in proteins, triggering the inhibition of synthesis of metabolic enzymes and protein (Schachtman and Liu, 1999). High NaCl accumulation at the root zone reduces the potentiality of water that restricts the extraction of water and create osmotic stress. The high concentration of salt decreased conductivity of stomata which restricts the inﬂux of leaf CO_2_ and creates the adverse chloroplast’s CO_2_/O_2_ ratio (Remorini et al., 2009). This impairs the photosynthetic apparatus and electron transport, leading to reduced productivity and photosynthesis (Deinlein et al., 2014). Rubisco enzymes enhance the oxygenase activity due to a deficiency of internal CO_2_ concentration and augmented rate of photorespiration, resulting formation of O_2_^•-^ (Hsu and Kao, 2003) and generation of greater H_2_O_2_ in the leaf tissue (Hernandez et al., 2000).

Plants have distinct pathways to detoxify ROS through a range of enzymes, compatible solutes (CS) or non-enzymatic secondary metabolites and antioxidants solely or in combination to counterbalance osmotic stress (Ashraf and Foolad, 2007; Gill and Tuteja, 2010). To adjust salt-induced osmotic stress, plants accumulate compatible solutes including soluble protein, proline, soluble sugars, antioxidants like ascorbate (AsA) (Munné-Bosch, 2005; Rai et al., 2013; Talbi et al., 2015). These compounds increase water absorption by reduction of the osmotic potential of cytoplasm (Puniran-Hartley et al., 2014). Antioxidant Enzymes including peroxidase (GPOX), superoxide dismutase (SOD), catalase (CAT), and AsA peroxidase (APX) that detoxify ROS (Gill and Tuteja, 2010).

Currently, researches are more attentive to the exploration of the defense system of antioxidants in leafy vegetables under salt stress. There is no information on the salt tolerance mechanism of *A. tricolor* genotypes regarding the regulation of free oxygen radicals quenching pathway through the antioxidative defense. In our previous studies (Sarker et al., 2014; Sarker et al., 2015a; Sarker et al., 2015b; Sarker et al., 2016; Sarker et al., 2017; Chakrabarty et al., 2018; Sarker et al., 2018a; Sarker et al., 2018b; Sarker et al., 2018c; Sarker and Oba, 2019a; Sarker and Oba, 2019b; Sarker and Oba, 2019c; Sarker and Oba, 2020a; Sarker and Oba, 2020b; Sarker and Oba, 2020c; Sarker and Oba, 2020d; Sarker and Oba, 2020e; Sarker et al., 2020a; Sarker et al., 2020b; Sarker et al., 2020c; Rashad and Sarker, 2020) we screened antioxidants enrich and high yield potential genotypes. Based on physiological and morphological traits, we again screened selected antioxidant enriched and high yield potential genotypes against salinity stress and identified both tolerant and susceptible genotypes in our earlier experiment (Data not published). In selected genotypes, we also observed tremendous augmentation of ascorbate under drought (Sarker and Oba, 2018a; Sarker and Oba, 2018b) and salinity stress (Sarker and Oba, 2018c; Sarker et al., 2018; Sarker and Oba, 2019d) and a tremendous increment of APX with drought severity (Sarker and Oba, 2018d; Sarker and Oba, 2018e). The increment of non-enzymatic antioxidants such as ascorbate and antioxidant enzyme APX is the sign of tolerance. Furthermore, there is scarce information regarding the response of salinity stress to anatomical, physiological, non-enzymatic, and enzymatic antioxidants of *A. tricolor*. It was hypothesized that the response of growth, anatomical, physiological, non-enzymatic, and enzymatic antioxidants may be differential in salinity stress tolerance and sensitivity to *A. tricolor*. Therefore, present investigations were aimed to better understand and elucidate growth, anatomical, physiological, non-enzymatic and enzymatic antioxidative defense pathways regarding salt-tolerant by comparing selected *A. tricolor* genotypes, (i) which component(s) of antioxidant defense system responsible for ROS detoxification and salt tolerance in *A. tricolor*? (ii) Whether the component(s) showed differential roles in tolerant and sensitive *A. tricolor* varieties.

## Materials and Methods

### Plant Materials and Experimental Conditions

We screened *A. tricolor* varieties against salinity stress based on physiological and morphological traits and identified both susceptible and tolerant varieties in our earlier experiment (Data not published). Two *A. tricolor* varieties, VA14 (salt tolerant) and VA3 (moderately salt sensitive) were used in this investigation. The seeds of two varieties were collected from the Department of Genetics and Plant Breeding and grown at Bangabandhu Sheikh Mujibur Rahman Agricultural University, Bangladesh (AEZ-28, 24°23 ′ North latitude, 90°08 ′ East longitude, and 8.4 m.a.s.l.). During rainfall events, the trial area was protected from rain by rain shelter covering, and otherwise, plants were exposed to ambient field conditions. Twenty-four plastic pots (22 cm × 60 cm × 40 cm) were filled up with sandy loam soil. The seeds were sown in pots maintaining the spacing of 20 cm × 5 cm rows and plants, respectively. The factorial experiment comprised of two treatments (salinity and variety) with four replications following a randomized complete block design (RCBD). N:P_2_O_5_:K_2_O were applied @ 92:48:60 kg ha^−1^, respectively as a split dose. The first dose, in pot soil, N:P_2_O_5_:K_2_O were applied @ 46:48:60 kg ha^−1^, respectively, and the second dose, at 10 DAS, N:P_2_O_5_:K_2_O were applied @ 46:0:0 kg ha^−1^, respectively. During the experimentation period, the relative humidity, day length, and average day/night temperatures were 74%, 12 h, and 25/21°C, respectively. We grouped one variety into 4 sets and 3 salinity treatments of 100 mM, 50 mM, and no saline water (NS) or control. Freshwater was provided as irrigation up to 25 DAS (days after sowing) for vigorous growth and proper establishment of seedlings. At 26 DAS, we imposed salinity treatment. Once a day, saline water (100 mM, 50 mM) and freshwater were provided in respective pots. Up to 55 DAS, salinity treatments were continued. At the edible stage (55 DAS) the leaves were harvested. At midday, quantification of plant parameters was sampled from young fully emerged leaves. All the traits were estimated in four replicates.

### Plant Growth and Leaves Anatomy Measurements

Five plants were harvested at 55 DAS for measuring specific leaf area and total biomass. A leaf area meter was utilized to estimate the leaf area per plant. The samples were dried at 70°C to obtain total plant and leaves dry mass. The total biomass, shoot dry weight, and dry weight of leaf was measured. The total leaf area of the plant was divided by the dry weight of leaf to measure the specific leaf area. For light microscopy observation, specimens were embedded in Quetol 651 resin. We made 1 µm thick cross-sections from a leaf using an ultramicrotome. Cross-sections were stained with toluidine blue O (1%) in borax. Finally, a Nikon light microscope was used to examine these cross-sections. All the leaf anatomy traits were estimated from 10 specimens randomly taken.

### Chlorophyll and Total Carotenoid Content Estimation

Carotenoids and chlorophylls were calculated through the extraction of leaves in acetone (80%) (Sarker and Oba, 2018a; Sarker and Oba, 2020a). A Hitachi spectrophotometer (Japan) was used to read the absorbance at 470, 646, and 663 nm for carotenoids, chlorophyll *b*, and chlorophyll *a*, respectively. Data were estimated as μg chlorophyll per g and mg total carotenoid per 100 g fresh weight (FW), respectively.

### Estimation of the Relative Water Content of Leaf

The relative water content of leaf (RWC) was measured following the method of Ogbaga et al. (2014). In each replication, leaves (fully expanded) of 3 plants were used for estimation of the relative water content of leaf (RWC). Ten mm in diameter 3 leaf discs were punched using a cork borer from the interveinal area of each plant. Replication-wise fresh weight (FW) of average discs was measured immediately. The weighed leaf discs were placed in distilled water under dim illumination at 20°C for 4 h for complete hydration. Turgid mass (TW) of blotted leaf discs were taken to estimate water uptake. The leaf discs were dried at 70°C for 2-4 d to determine the dry mass (DW). RWC was estimated as (FW - DW)/(TW - DW) × 100.

### Determination of Leaf Malondialdehyde and H_2_O_2_

2-thiobarbituric acid (TBA) was utilized to estimate malondialdehyde (MDA) following methods of Zhao et al. (2006). In a mortar and pestle, fresh *A. tricolor* leaf samples (1 g) were grounded in 5 mL TBA (0.6%) and trichloroacetic acid (TCA) (10%). At a temperature of 100°C, we heated the mixture for 15 min. After cooling it in ice, we centrifuged the mixture at 5000 rpm/min for 10 min. The absorbance was taken at 450, 532, and 600 nm. The content of MDA was determined on a fresh weight basis as follows:

μmol  MDA  g−1  FW  = 6.45 (OD532 - OD600)  -  0.560OD450,and  finally  data  were  recorded  as μmol  per gram  fresh  weight (μmol g−1 FW).

The KI method (Alexieva et al., 2001) was utilized to estimate hydrogen peroxide. Exactly 0.5 mL trichloroacetic acid (TCA) (0.1%), leaf extract supernatant, 0.5 mL potassium phosphate buffer (100 mM), and 2 mL reagent 1 mL KI (w/v double-distilled water) were utilized in the reaction mixture. A blank probe was made using TCA (0.1%) in the absence of leaf extract. The reaction mixture was allowed to stand in the dark for one h to read the absorbance at 390 nm. A standard curve was used to estimate the amount of hydrogen peroxide. Finally, data were measured as μmoles per gram fresh weight (μmol g^-1^ FW).

### Electrolyte Leakage Determination

The method of Lutts et al. (1995) was followed to determine electrolyte leakage (EL). Six randomly selected plants (4 leaves (mature) in each plant) per treatment were harvested and cut into 1 cm segments. The samples were washed 3 times using distilled water to remove surface contamination. The samples were each placed in stoppered vials with 10 mL distilled water and incubated at 25°C on a shaker (100 rpm) for 24 h. After incubation, we measured the electrical conductivity of the bathing solution (EC1). The solution was cooled at 25°C. We placed the same samples in an autoclave for 20 min at 120°C and a reading of the EC (EC2) was measured. The EL was measured as EC1/EC2 and expressed as the percentage.

### Estimation of Compatible Solutes and Non-Enzymatic Antioxidants

#### Determination of Soluble Sugar Content

Phenol-sulfuric acid method and D-(+)-glucose as a standard (DuBois et al., 1956) were utilized to estimate soluble sugar content. A spectrophotometer was used to read the absorbance at 490 nm.

#### Determination of Soluble Protein Content

Soluble proteins were estimated using the dye-binding method and bovine serum albumin as a standard (Bradford, 1976). A spectrophotometer was used to take the absorbance at 595 nm.

#### Estimation of Leaf Proline Content

Sulfosalicylic (3%) and extraction buffers ninhydrin (Bates et al., 1973) were utilized to estimate proline from freeze-dried leaves. Sulfosalicylic acid (3% w/v) was utilized to extract 0.04 g dried leaves. The extract was centrifuged for 10 min at 3000 × g. Four hundred μL of the mixture of reagent (1.25 g ninhydrin, 20 mL phosphoric acid, and 30 mL glacial acetic acid) was mixed with a 200 μL supernatant aliquot and heated in sealed test tubes at 100°C for 1 h. The mixture was then cooled. Exactly 4 mL of toluene was added to the mixture of every sample. A Hitachi spectrophotometer (Japan) was used to estimate proline content at 520 nm. The data was recorded as μmol per g dry weight (μmol g^-1^ DW).

#### Determination of Ascorbate (ASA) and Dehydroascorbate (DHA)

DHA and ASA of leaves were estimated using the method of Ma et al. (2008) with slight modifications. On the ice, 8 mL TCA (5% w/v) was utilized to homogenize 0.5 g dried leaf powder. The mixture was centrifuged for 10 min at 4°C at 10,000 × g. The supernatant was utilized immediately for analysis. In 1 mL TCA (10% w/v), 800 μL of 42% (w/v) orthophosphoric acid, 800 μL 65 mM 2, 2-dipyridyl in 70% (v/v) ethanol, and 400 μL 3% (w/v) ferric chloride reaction mixture 0.8 mL supernatant was added. The reaction was incubated at 42°C for 1 h. We read absorbance at 525 nm. To estimate ASA content, a standard curve generated with known ASA concentrations was used. The extract was incubated for 15 min at 42 °C with 10 mM dithiothreitol (DTT) in phosphate buffer (0.2 mM) at pH 7.4 (0.2 mL) to measure total ASA. Exactly 0.2 mL *N*-ethylmaleimide (0.5% w/v) was utilized to stop the reaction. DHA levels were estimated as DHA = (ASA_t_ - ASA).

#### Extraction of Samples for TAC Analyses

Amaranth leaves were harvested at thirty days old. The leaves were dried in an open shady place. Forty mL aqueous methanol (90%) was utilized to extract the sample (1 g) in a capped bottle (100 mL). A shaking water bath (T-N22S, Japan) was utilized to extract leaf samples for 1 h. Exactly 0.45 µm ﬁlter (MILLEX^®^-HV, USA) was utilized to ﬁlter the mixture. After centrifugation for 15 min at 10,000 × g, total antioxidant activity was estimated from the filtered extract.

#### Estimation of Total Polyphenols (TPC)

Extraction and quantification of total polyphenols were carried out according to Sarker and Oba (2020c). In a water bath, samples (25 mg) were added to 2.5 mL HCl (1.2 M) containing methanol (90%) and allowed to stand for 2 h at 90°C. With readjusting the volume (2.5 mL), the leaf extract was centrifuged at 7500 rpm for 20 min. The leaf extracts (100 µL) were added to the Folin-Ciocalteau reagent (2 N, 50 µL) and allowed to stand for 5 min. Then, Na_2_CO_3_ (400 µL, 2 N) and water (1 mL) were added to the mixture. The leaf extracts were incubated for 90 min at 37°C. Finally, it was removed to a microplate (ﬂat bottom). In a microplate reader, the absorbance was detected at 740 nm. We estimated the results equivalent to gallic acid (GAE) standard µg g^-1^ of dw.

#### Estimation of Total Favonoids (TFC)

Total ﬂavonoids were quantiﬁed and extracted following the method of Sarker and Oba (2020d). In water, leaves (100 mg) were added to methanol (5 mL, 50%) and allowed to stand for 1 h with ultrasound. The leaf extracts were centrifuged at 13,000 × g for 10 min at 4°C. Then we recovered the supernatants. The extracts (400 µL) were added to NaNO_2_ (60 µL, 5%), water (500 µL), and AlCl3 (140 µL, 10%) and allowed to stand for 10 min. Then, NaOH (400 µL, 1 mM) was added to the mixture. The leaf extracts were incubated for 10 min at room temperature. Finally, it was removed to a microplate (ﬂat bottom). In a microplate reader, the absorbance was detected at 500 nm. We estimated the results equivalents to rutin (RE) standard µg g^-1^ of dw.

#### Determination of Total Antioxidant Capacity (TAC)

Diphenyl-picrylhydrazyl (DPPH) radical degradation method (Sarker and Oba, 2018a; Sarker et al., 2020b) was used to estimate the antioxidant activity. We added 1 mL DPPH solution (250 µM) to 10 µL extract (in triplicate) in a test tube. After adding 4 mL distilled water the extract was placed in the dark for 30 min. A Hitachi spectrophotometer (Japan) was utilized to measure the absorbance at 517 nm. ABTS^+^ assay was performed following the method of Sarker and Oba (2018a). To prepare two stock solutions separately ABTS^+^ solution of 7.4 mM and potassium persulfate of 2.6 mM were used. We mixed both solutions in equal proportion to prepare the working solution at room temperature. The working solution was allowed to react in the dark for 12 h. One hundred fifty μL extract was added to 2.85 mL of ABTS^+^ solution and allowed to react in the dark for 2 h. For the preparation of the solution, one mL of ABTS^+^ solution was mixed with sixty mL of methanol. A Hitachi spectrophotometer (U1800, Tokyo, Japan) was utilized to take the absorbance against methanol at 734 nm. The inhibition (%) of DPPH and ABTS^+^ corresponding with control was used to determine antioxidant capacity using the equation as follows:

Antioxidant activity (%)=(Abs. blank- Abs. sample/Abs. blank)×100

Where, Abs. blank is the absorbance of the control reaction [10 µL methanol for TAC (DPPH), 150 μL methanol for TAC (ABTS^+^) instead of leaf extract], and Abs. sample is the absorbance of the test compound. Trolox was used as the reference standard, and the results were expressed as Trolox equivalent μg g^-1^ dw.

### Determination of Activities of Antioxidant Enzymes

Leaf samples (1 g) were frozen in nitrogen (liquid) and subsequently grounded in extraction buffer (10 mL). Extraction buffer was prepared from 0.1 M buffer (phosphate pH 7.5) including EDTA (0.5 mM) for estimation of CAT, GPOX, SOD, and ascorbate and (1 mM) for estimation of APX. The extract was passed through a cheesecloth filter (4 layered). Then, these were centrifuged at 15000 × g for 20 min at 4°C. The activity enzymes were assessed using the supernatant. A temperature of four °C was maintained to carry out all steps of the preparation of the enzyme extract.

The reduction of photochemical of nitroblue tetrazolium (NBT) through inhibition of enzyme was assessed to determine total SOD (EC 1.15.1.1) activity (Dhindsa et al., 1981). Exactly 3 mL reaction mixture was added to 0.1 mL riboflavin (2 mM) to start the reaction. The reaction mixture was prepared by mixing enzyme extract (0.1 mL) with methionine (13.33 mM), 50 mM buffer (phosphate, pH 7.8), sodium carbonate (50 mM), 75 µM NBT, and EDTA (0.1 mM). Two 15 W fluorescent lamps were placed in the tubes for 15 min. We read the absorbance at 560 nm. The amount of enzyme that reduced the reading of absorbance to 50% compared to tubes lacking enzyme was estimated as enzyme activity (1 unit).

Guaiacol peroxidase activity (GPOX, EC 1.11.1.7) was estimated from the increment of absorbance because of the formation of tetra-guaiacol at 470 nm. The activity of the enzyme was estimated based on the coefficient of extinction of tetra-guaiacol (an oxidation product of enzyme), ϵ = 26.6 mM^-1^ cm^-1^ (Castillo et al., 1984). The reaction mixture was prepared by mixing enzyme extract (0.1 mL) with phosphate buffer (50 mM, pH 6.1), H_2_O_2 (_2 mM), and guaiacol (16 mM). Distilled water was used to dilute the mixture with a final volume of 3.0 mL. The activity of the enzyme was estimated as micromoles tetra-guaiacol min^-1^ mg^-1^ protein.

Catalase was estimated from the measurement of the disappearance of H_2_O_2_ (Aebi, 1984). The reaction mixture was prepared by mixing 0.5 mL H_2_O_2_ (75 mM), 1.5 mL buffer (phosphate 0.1 M, pH 7), and enzyme extract (50 µL, diluted). The decrement in absorbance was observed at 240 nm for 1 min. The activity of the catalase enzyme was estimated from the amount of decomposed H_2_O_2_.

Ascorbate peroxidase (EC 1.11.1.11) was assessed by estimating the decrement in optical density because of ascorbate at 290 nm (Nakano and Asada, 1981). Exactly 3 mL of reaction mixture was prepared by mixing 50 mM buffer (potassium phosphate, pH 7.0), 0.1 mL H_2_O_2_ (0.1 mM), EDTA (0.1 mM), ascorbate (0.5 mM), enzyme extract (0.1 mL) and water. The decrement of absorbance was assessed spectrophotometrically. A standard curve was used to measure the activity by estimating the decrement of ascorbate.

### Estimation of Na and K Content

Dried leaf powder of *A. tricolor* was analyzed for measurement of K and Na content following the nitric-perchloric acid digestion method according to Sarker and Oba (2018a) at the wavelength of 766.5 nm (K) and 589.0 nm (Na) using Hitachi atomic absorption spectrophotometer (AAS) (Japan).

### Statistical Analysis

The results were expressed as mean and standard deviation of four separate replicates. A Statistix 8 software was used to analyze the data (Hasan-Ud-Daula and Sarker, 2020). The means were compared by the DMRT test at a 1% probability level. Figures were prepared using the Microsoft Excel program.

## Results

### Salinity Effects on Plant Growth and Leaves Anatomy

**Figure 1:**
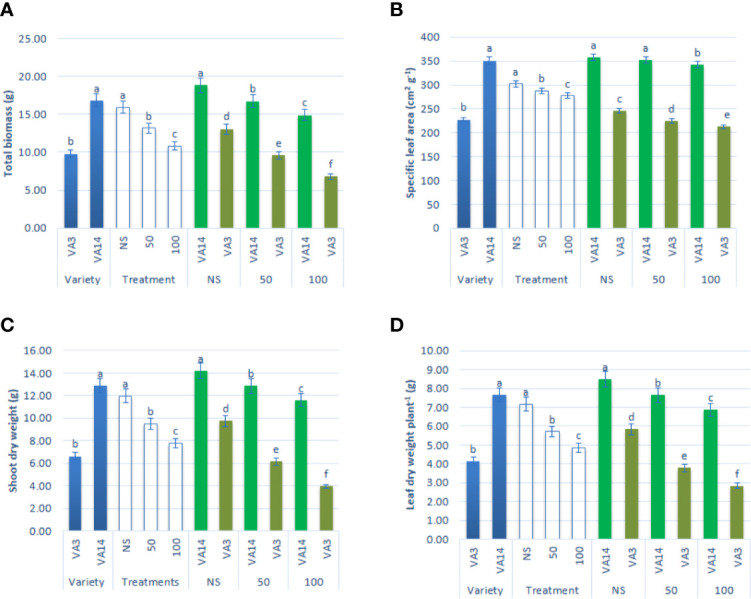
Effect of different salinity levels on growth parameters: total biomass **(A)**, specific leaf area **(B)**, shoot dry weight **(C)** and leaf dry weight **(D)** in *A. tricolor*. Values are means of four replicates and different letters are differed signiﬁcantly by Duncan Multiple Range Test (P < 0.01).

The major growth parameters, such as leaf dry weight per plant, specific leaf area, total biomass, and shoot dry weight of both varieties were significantly reduced both at 50 mM and 100 mM salt concentrations compared with those at control or no saline (NS) condition. The decline in leaf dry weight per plant, specific leaf area, total biomass, and shoot dry weight were pronounced in VA3 in comparison to VA14 across salinity stress (**Figures 1A–D**).

VA14 had higher whole leaf thickness, the ratio of the thickness of palisade tissue and the thickness of the spongy tissue, the thickness of the palisade tissue, larger vessel frequency, and narrower vessel lumen compared to VA3 (**Table 1**). The leaf anatomical parameters demonstrated no significant differences between 50 mM salt stress treatment and control. In contrast, the leaf anatomical parameters were increased and vessel lumen diameter was decreased at severe salt stress treatment (100 mM) (**Table 1**).

**Table 1:** Effect of genotype and salinity stress on anatomical properties of *A. tricolor* leaves.

	Leaf thickness (µm)	Thickness of upper epidermis (µm)	Thickness of lower epidermis (µm)	Thickness of palisade tissue (TPT) (µm)	Thickness of spongy tissue (TST) (µm)	Ratio of TPT/TST (µm)	Vessel lumen diameter (µm)
**Genotype (G)**							
VA14	100. 73 ± 2.25a	16.83 ± 0.12a	13.06 ± 0.08a	39.12 ± 0.52a	30.38 ± 0.35a	1.29 ± 0.08a	12.84 ± 0.16b
VA3	90.32 ± 1.46b	16.48 ± 0.38a	12.46 ± 0.14a	32.72 ± 0.45b	29.66 ± 0.42a	1.10 ± 0.06b	17.28 ± 0.21a
**Salinity treatment (S)**							
Control	87.89 ± 1.15b	14.32 ± 0.48b	11.25 ± 0.12b	31.62 ± 0.37b	28.55 ± 0.24b	1.11 ± 0.05b	16.54 ± 0.24a
50 mM	88.92 ± 1.32b	15.12 ± 0.33b	12.36 ± 0.09b	33.38 ± 0.28b	29.82 ± 0.31b	1.12 ± 0.04b	16.28 ± 0.18a
100 mM	109.78 ± 1.06a	20.54 ± 0.21a	14.67 ± 0.08a	42.75 ± 0.22a	31.68 ± 0.27a	1.35 ± 0.06a	12.36 ± 0.22b
G	*	ns	ns	*	ns	*	*
S	*	*	*	*	*	*	*
G × S	ns	ns	ns	ns	ns	ns	ns

In a column, mean values with different letters are differed signiﬁcantly by Duncan multiple Range Test, ns, non-significant, * = significant, (P < 0.01), (n = 10).

### Salinity Effects on Photosynthetic Pigment Content

**Figure 2:**
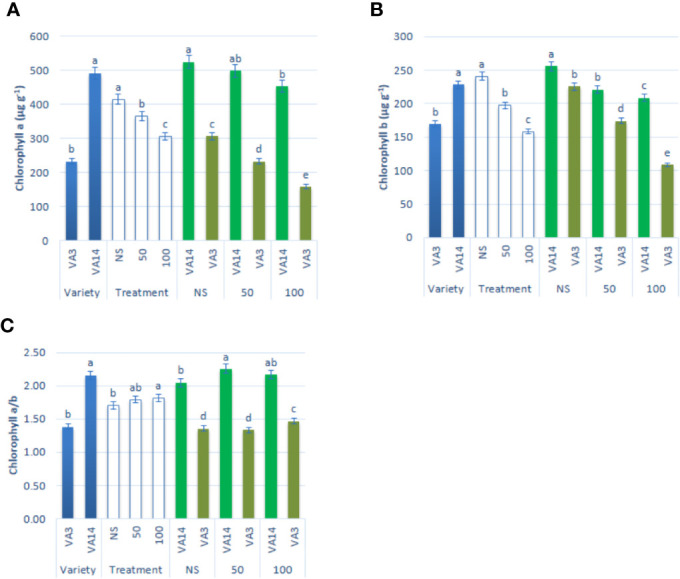
Effect of different salinity levels on photosynthetic pigment biosynthesis: chlorophyll *a* **(A)**, chlorophyll *b* **(B)** and chlorophyll *a/b* **(C)** in *A. tricolor*. Values are means of four replicates and different letters are differed signiﬁcantly by Duncan Multiple Range Test (P < 0.01).

Chlorophyll *a* and chlorophyll *b* contents were significantly and remarkably declined with the increment of salinity level of 50 mM and 100 mM (**Figures 2A, B****)** compared to the control condition. The decline of chlorophyll *a* and chlorophyll *b* contents was much pronounced in VA3 in all the salinity treatments. However, VA14 showed a static ratio in 50 mM but a significant decline in 100 mM salt concentration compared with control. Chlorophyll *a*/*b* ratio significantly differed between variety and salinity treatment (P < 0.01, **Figure 2C**). Chlorophyll *a*/*b* ratio of VA14 was significantly increased in 50 mM salt treatment and slightly reduced in 100 mM salt treatment while VA3 showed a static ratio in 50 mM but a significant increase in 100 mM salt concentration compared with control (**Figure 2C**).

### Impact of Leaf Relative Water Content, Hydrogen Peroxide, Lipid Peroxidation, and EL on Salinity

**Figure 3:**
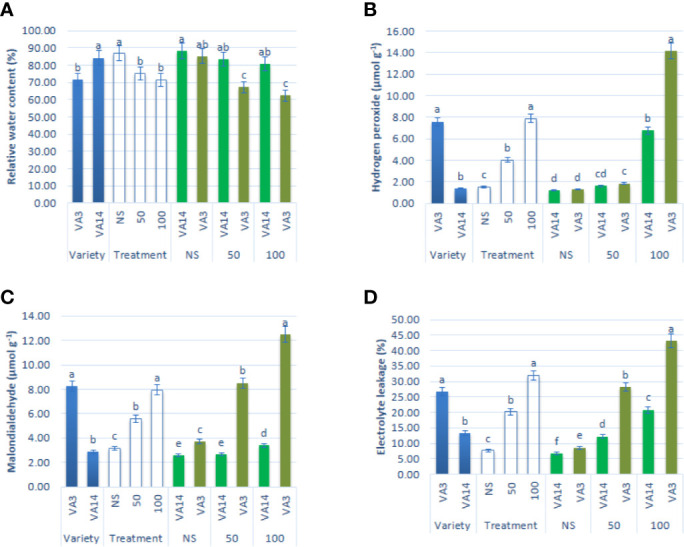
Effect of different salinity levels on ROS marker: relative water content (RWC, **A**), hydrogen peroxide (H_2_O_2,_ **B**), malondialdehyde content (MDA, **C**), and electrolyte leakage (EL, **D**), in *A. tricolor*. Values are means of four replicates and different letters are differed signiﬁcantly by Duncan Multiple Range Test (P < 0.01).

Leaf RWC showed a significant reduction of up to 50 mM, while it showed a static ratio in 50 mM and 100 mM salinity stress conditions (**Figure 3A**). H_2_O_2_, MDA, and EL content sharply increased with the severity of salinity stress (**Figures 3B–D**). Hydrogen peroxide, MDA, and EL were the highest in VA3 and the lowest in VA14. VA3 had the highest increment of MDA, EL, and hydrogen peroxide compared to VA14 at 50 mM and 100 mM salt treatment (**Figures 3B–D**).

### Effect of Salinity on Compatible Solutes and Non-Enzymatic Antioxidants

**Figure 4:**
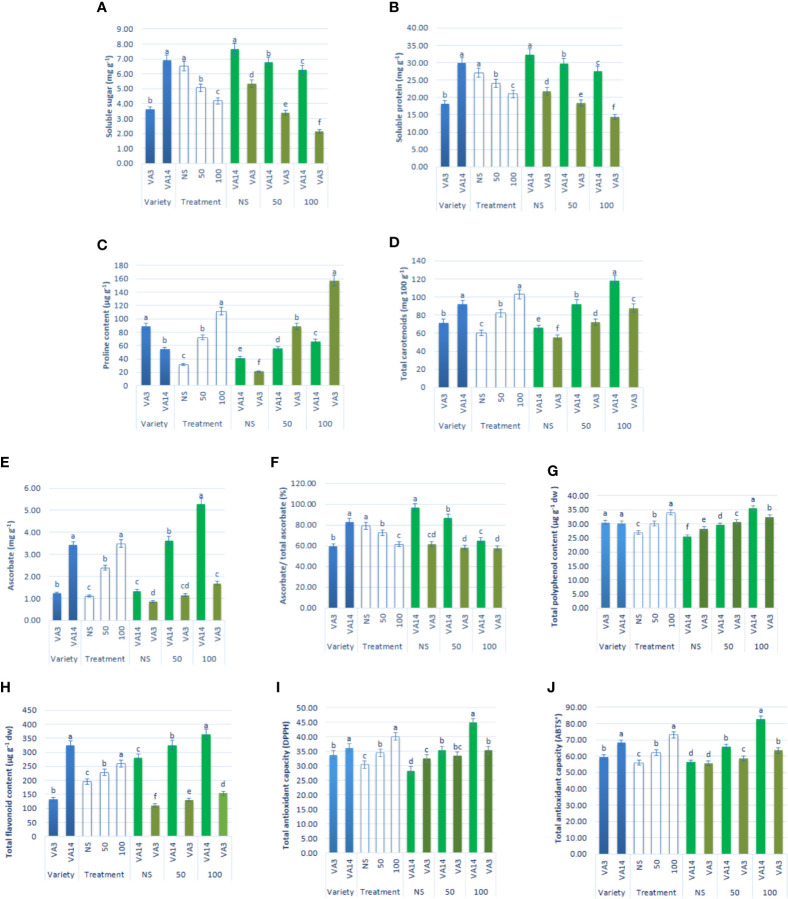
Effect of different salinity levels on compatible solutes and non-enzymatic antioxidants: soluble sugar **(A)**, soluble protein **(B)**, proline content **(C)**, total carotenoid **(D)**, reduced ascorbate (AsA, **E**), ascorbate redox status (reduced/total ascorbate, **F**), total polyphenol content (GAE µg g^-1^ dw, **(G)**, total flavonoid content (RE µg g^-1^ dw, **(H)**, total antioxidant capacity (DPPH) (TEAC µg g^-1^ dw, **(I)** and total antioxidant capacity (ABTS^+^) (TEAC µg g^-1^ dw, **(J)** in *A. tricolor*. Values are means of four replicates and different letters are differed signiﬁcantly by Duncan Multiple Range Test (P < 0.01).

Soluble sugar and soluble protein content sharply declined at 50 mM and 100 mM salt stress, compared to the control condition, while the decline of soluble sugar and soluble protein content of VA3 was greater compared with VA14 (**Figures 4A, B**). Proline accumulation remarkably increased by 1.33 and 2.58fold, respectively, at 50 mM and 100 mM salt stress, in comparison to the control condition. VA3 had the highest proline content of all salt stress treatments in comparison to VA14 (**Figure 4C**). Similarly, total carotenoids progressively increased by 37% and 72% respectively, at 50 mM and 100 mM salt stress, in comparison to the control condition. VA14 had the highest total carotenoids content of all salt stress treatments in comparison to VA3 (**Figure 4D**). There was a signiﬁcant and sharp increase in ascorbate (AsA), TPC, TFC, TAC (DPPH) and TAC (ABTS^+^) under 50 mM (1.18fold, 15%, 16%, 25% and 16%) and 100 mM (2.17fold, 39%, 30%, 58% and 47%) salinity stress across over varieties (**Figures 4E–J**). However, an increase in ascorbate, TPC, TFC, TAC (DPPH), and TAC (ABTS^+^) in VA14 was the highest and most highly pronounced compared to VA3 of all salt stress conditions (**Figures 4E–J**). In VA14, ascorbate redox status (AsA/total AsA) was signiﬁcantly and sharply decreased under 50 mM (18.14%) and 100 mM (28.97%) salinity stress, however, VA3 showed a static ascorbate redox status across salinity stress (**Figure 4F**).

### Effect of Salinity on Antioxidant Enzyme Activity

**Figure 5:**
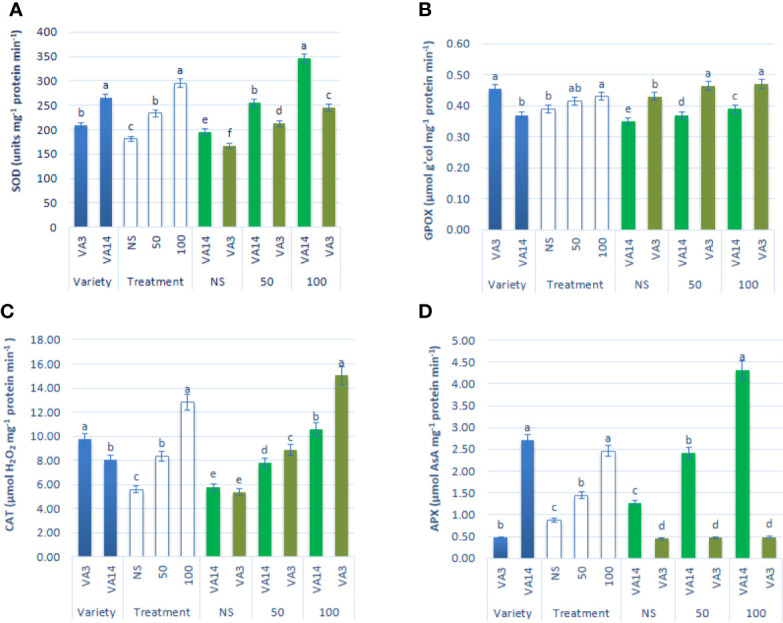
Effect of different salinity levels on antioxidant enzyme activities: superoxide dismutase (SOD, **A**), peroxidase (GPOX, **B**), catalase (CAT, **C**) and ascorbate peroxidase (APX, **D**), in *A. tricolor*. Values are means of four replicates and different letters are differed signiﬁcantly by Duncan Multiple Range Test (P < 0.01).

SOD activity was the highest in VA14 and the lowest in VA3. SOD activity was stimulated with the severity of salinity stress at 50 mM (30%) and 100 mM (63%) salt stress condition in comparison to the control condition in both varieties, however, the increment of SOD activity in VA14 was greater in comparison to VA3 at all salinity levels (**Figure 5A**). VA3 showed greater GPOX and CAT activity compared to VA14. Like SOD activity, GPOX and CAT activity had significant increases at 50 mM (8% and 49%) and 100 mM (11% and 129%) salt stress condition compared with a control condition in the varieties studied, whereas VA3 demonstrated the greater increase in comparison to VA14 across salinity stress (**Figures 5B, C**). VA14 had a greater APX activity than VA3. There was no significant difference in APX activity in VA3 under control, 50 mM and 100 mM salt concentration, while VA14 exhibited the greatest dramatic increase in APX activity at 50 mM (79%) and 100 mM (240%) salt stress in comparison to the control condition (**Figure 5D**).

### Impact of Salinity on Na^+^ and K^+^ Content

**Figure 6:**
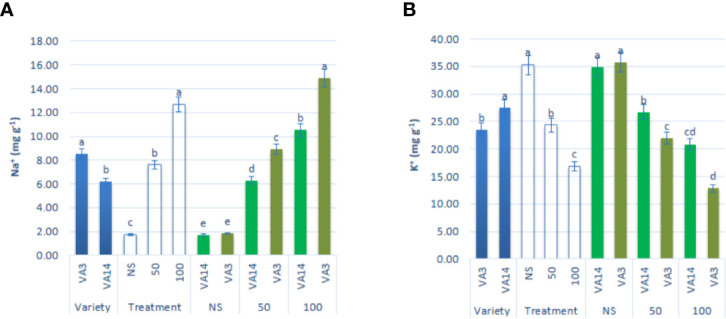
Effect of different salinity levels on Na^+^ **(A)**, and K^+^ **(B)** ion content in *A. tricolor*. Values are means of four replicates and different letters are differed signiﬁcantly by Duncan Multiple Range Test (P < 0.01).

Na^+^ content signiﬁcantly and sharply augmented with the increase in salinity stress showing the order: control < 50 mM < 100 mM, 3.32, and 6.22fold, respectively. However, the response of VA3 was much greater than the response of VA14 at all salt stress treatment (**Figure 6A**). In contrast, K^+^ accumulation was drastically reduced from control to 100 mM salt stress in the progressive order: control > 50 mM > 100 mM, 32%, and 53% respectively, although the reduction was much higher in VA3 compared to VA14 (**Figure 6B**).

## Discussion

Inclusive findings of the current study proposed that *A. tricolor* is a tolerant crop to salinity stress. Complete plants stand in the sensitive variety at severe salt stress (100 mM NaCl) made it possible to compare and contrast the responses of tolerant and sensitive varieties. We screened and select a tolerant and susceptible variety based on physiological and morphological traits against salinity stress to explain the major physiological, anatomical, and antioxidative mechanisms of the defense systems involved. Mechanisms played a significant and differential role in the sensitive and tolerant varieties. We discussed these mechanisms elaborately in the following sections.

Studied growth traits significantly reduced in both varieties at 50 mM and 100 mM salt stress, in comparison to the control condition, indicating that salt stress suppressed the growth of both varieties. Growth reduction in VA3 was signiﬁcantly pronounced than that in VA14 under both salt stress conditions i.e., VA14 showed better adaptation compared to VA3. Menezes et al. (2017) observed a decrease in leaf dry mass, stem dry mass, root dry mass, total dry mass and leaf area at 25, 50, 75, and 100 mM of NaCl salt stress in *A. cruentus*. Odjegba and Chukwunwike (2012) noted a drastic decline in growth in *Amaranthus hybridus*. Omami et al. (2006) noted a decline in plant height, leaf area, specific leaf area, shoot dry mass, and root dry mass under different salt concentrations in *A. tricolor*, Accession ‘83, *A. hypochondriacus*, and *A. cruentus*. The salinity-induced decline in growth may be due to the creation of osmotic stress that inhibits transport and absorption of water. This inhibition leads to hormones-induced sequential reactions that reduce the opening of stomatal, assimilation of CO_2_, photosynthetic rate, (Odjegba and Chukwunwike, 2012; Menezes et al., 2017). The diversion of energy from growth to the homeostasis of salinity stress and a reduction in carbon gains may be another reason for growth decline (Atkin and Macherel, 2009). Mostly two mechanisms, such as tolerance and avoidance are involved in the adjustment of plants to salinity stress (Hukin et al., 2005; Yue et al., 2006). In avoidance mechanism, plants frequently produce thickening leaf lamellae, high frequency of vessel, and narrow vessel lumen, that can avoid embolism, reduce water losses, and balance water transport under stress conditions (Beniwal et al., 2010; Cao et al., 2014). We observed an increment in all the leaf anatomical parameters and a decrease in vessel lumen diameter under the more severe salt stress treatment (100 mM), establishing well acclimation to severe stress of both varieties. VA14 had well adapted to salt stress due to its higher thickness of the palisade tissue, the ratio of the thickness of palisade tissue, whole leaf thickness, narrower vessel lumen, larger vessel frequency, and thickness of the spongy tissue in comparison to VA3. Within the two varieties studied, the salt-sensitive VA3 was observed to be more susceptible to salinity in comparison to the salt-tolerant VA14. In addition, it is known that salt stress may cause changes in leaf anatomy. For example, in Eugenia plants, NaCl leaves of 8 dS/m (equivalent to 88 mM NaCl) increase the percentage of palisade parenchyma and intercellular spaces and decreased spongy parenchyma (Acosta-Motos et al., 2015). These changes may ensure the diffusion of CO_2_ to the chloroplast efficiently in a reduced aperture of stomata (Acosta-Motos et al., 2017).

Salinity stress affected the uptake and metabolism of essential elements like N, P, K, S and Ca leading to significant alterations in the photosynthetic efficiency. The decline in pigment for photosynthesis under salt stress also linked with the oxidation of chlorophyll pigment through free radicals, interference of salt ions with pigment-protein complexes (Kato and Shimizu, 1985), chloroplasts disruption or increased activity of chlorophyllase enzyme responsible for the degradation of chlorophylls (Parida et al., 2004). Odjegba and Chukwunwike (2012) in *A. hybridus* reported a similar reduction of chlorophyll content under different salt concentrations. Shahbaz et al. (2013) reported a drought-induced decline in chlorophyll content in wheat. The concentration of chlorophyll in stressed tissues is the index of salinity tolerance (Lutts et al., 1996). Salt-induced tolerant species show augmented or static chlorophyll content, while chlorophyll content decline in the sensitive species. In plants, chlorophyll content is one of the key markers for the study of tolerance to salinity (Stepien and Johnson, 2009; Ashraf and Harris, 2013). In this study, VA14 containing more chlorophyll content than the VA3, in that regard, one of the reasons of the salt tolerance of VA14 genotype can be their higher chlorophyll contents. Both varieties exhibited a lower reduction in chlorophylls under salt stress. The presence of betalain pigments (betaxanthin and betacyanin) may act as an antioxidant and absorb radiation significantly to protect excessive harmful light in the chloroplasts. These findings were corroborative to the findings of Jain et al. (2015). In *Disphyma australe* they reported that greater betalains salt-induced plant exhibited more tolerant physiologically by the production of less H_2_O_2_, faster recoveries of PSII, and increased rates of assimilation of CO_2_, photochemical quenching, photochemical quantum yields, and water use efficiencies. Moreover, betalain (Betacyanin and betaxanthin) prevents the chloroplasts from salinity stress by scavenging the reactive oxygen species in thylakoids (Reddy et al., 2004), faster recoveries of PSII, photochemical quenching, and photochemical quantum yields Jain et al. (2015).

Relative water content is a useful variable that reﬂects the water status of the plant (Kadioglu et al., 2011). RWC is a key determinant of survival of leaves and metabolic activity that could be used as a parameter for comparing sensitive and tolerant plants at salinity stress (Sinclair and Ludlow, 1986). Both varieties exhibited the salt-induced decline of RWC at 50 mM and 100 mM salt concentration, however, VA3 demonstrated a drastic decline compared to VA14. Menezes et al. (2017) in *A. cruentus* and Odjegba and Chukwunwike (2012) in *A. hybridus* reported a similar reduction of RWC under different salt concentrations. Salinity stress reduces turgor pressure, and soil water availability inhibits the uptake of water, resulting in a decrease in RWC of leaves. VA14 exhibited the higher leaf RWC under salt stress conditions, which may be due to greater osmoregulation potential, augmented compatible solutes accumulation (except for proline) in comparison to VA3. Hence, VA14 demonstrated more efficiency in reducing the osmotic potential of cells by permitting roots to uptake water sufficiently for maintaining the turgidity of the cell in a hydrated condition. Enhanced electrolyte leakage (EL) and MDA are considered as a sign of membrane damage induced by salt stress (Feng et al., 2003). Odjegba and Chukwunwike (2012) in *A. hybridus* reported a similar raise of MDA under different salt concentrations. Our study revealed that augmentation of salinity severity progressively increased EL. Hence, EL could be utilized as an index to compare susceptibility and tolerance of a variety to stress. VA14 showed lower EL can be utilized as a tolerant variety in comparison to VA3. Lower EL could be associated with the tolerance to salinity stress. In addition, H_2_O_2_ and MDA augmented dramatically with salinity severity that damaged cellular membrane and increased EL in VA3. These findings agreed with the results of Mudgal et al. (2010).

Tolerance to salinity is a complicated trait. A series of mechanisms are involved to hinder the effects of the ion and osmosis under salt stress (Munns and Tester, 2008). Salt-induced osmotic effect results in the shortage of available water and often causes oxidative stress to cells that leads to the generation free oxygen radicals for channelizing excess reducing power produced because of decline in photosynthetic dark reaction (Hsu and Kao, 2003; Abogadallah et al., 2010) or mitochondrial respiration, photorespiration, and photosynthesis pathways (Sharma et al., 2013). Several studies on legumes and other vascular plants showed that the production of a large quantity of ROS causes the toxic effects of salt-induced oxidative stress (Hernandez et al., 2000; Mittova et al., 2004). Our study revealed that salt stress significantly augmented the production of H_2_O_2_, EL, and lipid peroxidation in *A. tricolor*. The sensitive variety (VA3) produced greater ROS, lipid peroxidation, and EL in comparison to the tolerant variety (VA14). From our results, it was clear that at similar salt stress, more ROS was accumulated in the leaf tissues of sensitive *A. tricolor*, whereas the tolerant variety accumulated comparatively lower ROS.

Maintaining the osmotic adaptation, compatible solute, such as soluble protein, soluble sugars, and proline were accumulated in plants. Proline accumulated in response to salt stress also act as low molecular weight cellular antioxidant that protects plants from salt-induced adverse effects through scavenging ROS, maintaining the integrity of the membrane, contributes to cellular osmotic adjustment and stabilization of enzymes/proteins (Ashraf and Foolad, 2007) Both the varieties demonstrated a significant increase in proline content at 50 mM and 100 mM salt stress, however, the augmentation was lower in the tolerant variety VA14 in comparison to the sensitive variety VA3. It indicated that sensitive variety VA3 generated more ROS that was detoxified by the overproduction of proline. Soluble sugar and soluble protein content were reduced in both varieties in response to salt stress severity, while the decline is more in VA3 compared to VA14. Menezes et al. (2017) reported a similar increase in proline content as well as a reduction in soluble carbohydrates and soluble protein content in *A. cruentus* at 50 and 100 mM salt concentrations. Little attention was made in carotenoids although carotenoids are capable to quench ^1^O_2_; and lipid peroxy-radicals, and to generate superoxide and prohibit lipid peroxidation under dehydrative forces. β-carotene plays a main protective role in photosynthetic tissue through protecting from oxidative damage, preventing the generation of singlet oxygen, and direct scavenging of triplet chlorophyll (Farooq et al., 2009). Lipophilic antioxidants, total carotenoids able to quench various forms of free oxygen radicals (Bartwal et al., 2013). Usually, the radiation between 400 and 550 nm was absorbed by total carotenoid and passed the captured energy to the chlorophyll (Durchan et al., 2012). As an antioxidant, they excited chlorophyll (Chl*) molecules to protect the formation of singlet oxygen in the photosynthetic apparatus, quench singlet oxygen to prohibit oxidative damage, and triplet sensitizer (3Chl*). Non-enzymatic antioxidants ascorbate and total carotenoid, TPC, TFC, TAC (DPPH), and TAC (ABTS^+^) play an important role in reducing oxidative stress and cellular ROS homeostasis regulation in plants (Ashraf, 2009). Our study revealed that carotenoid, TPC, TFC, and TAC were augmented in both varieties, whereas the increment was higher in VA14 compared to VA3. In the tolerant variety VA14, a dramatic rise in ascorbic acid, TPC, TFC, and TAC were observed, whereas negligible augmentation of ascorbic acid and TAC and lower increment of TPC, and TFC were noted in the sensitive variety VA3. It indicated that non-enzymatic antioxidants ascorbate, total carotenoid, TPC, TFC, TAC (DPPH), and TAC (ABTS^+^) played an important role in reducing oxidative stress and cellular ROS homeostasis regulation in tolerant variety VA14. Furthermore, the increased content of AsA signified the major contribution of the AsA–GSH cycle for quenching ROS (especially H_2_O_2_) in this amaranth species. The greater activities and transcripts of the AsA–GSH cycle were accumulated in salinity tolerant variety (Hernandez et al., 2000).

The study revealed salt stress-induced superoxide dismutase (SOD) activity in both varieties, whereas, greater SOD activity was observed in VA14 (tolerant variety) in comparison to VA3 (sensitive variety), indicating a major contribution of SOD in overall salt tolerance in *A tricolor* by enhancing dismutation reaction to convert superoxide radicals [SOR (O_2_^-.^)] to H_2_O_2_. These findings were agreed with the findings of Ben Amor et al. (2006) and Sekmen et al. (2007) where they associated the increment of SOD activity in the tolerant one. The plant scavenges ROS through mechanism coordinately, where first line defense is provided by SOD. Under unfavorable conditions, chloroplast generates superoxide mostly from the leakage of the respiratory and photosynthetic electron. SOD dismutases superoxide into H_2_O_2_. A variety of peroxidases including phenol peroxidase, glutathione peroxidase (GPX), and ascorbate peroxidase (APX) use various reducing agents and decompose H_2_O_2_ into the water (Asada, 1999). In contrast, primarily catalase (CAT) decomposes H_2_O_2_ that generates in the peroxisome due to photorespiration (Dat et al., 2000). H_2_O_2_ is decomposed by both peroxidases (APX and GPOX) and CAT, however generally, the affinity of decomposition of H_2_O_2_ by APX is much more pronounced than CAT (Abogadallah et al., 2010), indicating peroxidases may be associated with scavenging ROS at a lower concentration of H_2_O_2_, whereas CAT quenches much greater concentration of H_2_O_2_.

Odjegba and Chukwunwike (2012) in *A. hybridus* reported an increase in CAT and APX under different salt concentrations. Under salinity stress, VA3 (sensitive variety) showed a negligible increase in the activity of APX, whereas VA14 (tolerant variety) showed a pronounced increase in the activity of APX. It revealed that APX contributed as the principal scavenging enzyme in salt-induced *A. tricolor* at lower H_2_O_2._ Hence, APX regulated H_2_O_2_ in VA14 (tolerant variety) efficiently. Increase in ascorbate, and APX proves the active involvement of AsA–GSH cycle in scavenging ROS (especially H_2_O_2_) in tolerant *A. tricolor*. In contrast, the activity of GPOX and CAT showed remarkable and significant augmentation in both salt-induced varieties, whereas VA3 (sensitive variety) demonstrated the maximum augmentation in comparison to VA14 across all treatments. As GPOX and CAT showed greater augmentation in VA3 (sensitive variety) across all treatments in comparison to VA14 (tolerant variety), it is evident that both GPOX and CAT played a principal role to supplement APX activity in VA3 (sensitive variety) at a greater load of H_2_O_2_. In cowpea, salt stress-induced GPOX activity albeit CAT and SOD activity were non-significant (Cavalcanti et al., 2004). In barnyard grass, APX-GR and GPOX acted as the principal quencher of H_2_O_2_ at low H_2_O_2_ and regulated the ROS finely. Although CAT had the capability to scavenge H_2_O_2_ much more pronouncedly, it was unable to maintain the balance of ROS under salt stress (Abogadallah et al., 2010).

Non-speciﬁc ion uptake in salt-induced cells raises the concentration of Na^+^ ions. In salt-tolerant plants, two main mechanisms such as salt exclusion and sequestration are identified to maintain cytosolic Na^+^ level appropriately (Anower-Rokebul et al., 2013). In many plant species, physiologically the main mechanisms of salt tolerance are the uptake of selective K^+^ against Na^+^ (Asgari et al., 2012). In this investigation, both varieties showed a dramatic increase in Na^+^ and a drastic decline in K^+^ accumulation. Menezes et al. (2017) and Odjegba and Chukwunwike (2012) reported a similar increase in Na^+^ and reduction in K^+^ content at different salt concentrations in *A. cruentus* and *A. hybridus*, respectively. VA14 had the lowest Na^+^ uptake and the lowest reduction in K^+^ uptake under salt stress and enabled this to keep more RWC compared to VA3. Therefore, VA14 is capable of maintaining a high concentration of solute and is able to absorb more water and as a consequence have high RWC to adjust osmosis.

## Conclusion

In the current investigation, the salt sensitive and tolerant *A. tricolor* variety behaved differently under salt stress regarding growth, anatomical, physiological, ROS accumulation, enzymatic and non-enzymatic antioxidative defense mechanisms and attributes associated with tolerance to oxidative stress. Tolerant variety VA14 had higher RWC, photosynthetic pigment, lower EL, higher whole leaf thickness, the ratio of the palisade tissue thickness, and the thickness of the spongy tissue, narrower vessel lumen, larger vessel frequency, and the thickness of the palisade tissue and well adapted to salt stress. In the tolerant variety, lower ROS load might be due to greater defense of non-enzymatic and enzymatic antioxidants and antioxidants pool of cells. In this study, it seems that *A. tricolor* variety doesn’t necessarily require simultaneous induction of a full set of non-enzymatic and enzymatic antioxidant enzymes for salt tolerance, rather non-enzymatic antioxidant ascorbate, carotenoids, TPC, TFC, TAC, and antioxidant enzyme SOD and APX were observed to be the major H_2_O_2_ detoxifier in tolerant *A. tricolor* variety at a lower load of H_2_O_2_, whereas, GPOX and CAT activity was prominent at a greater load of H_2_O_2_ (in sensitive variety). SOD contributed to the dismutation of SOR in both tolerant and sensitive varieties, however; it had greatly contributed to dismutation of SOR in tolerant variety. An increase in SOD, ascorbate, and APX in tolerant *A. tricolor* indicated the major contribution of SOD and AsA–GSH cycle in quenching ROS (especially H_2_O_2_).

## Author Contributions

US initiated the research work and conceived the study. US performed the experiments. US performed biochemical analysis and statistical analysis. US drafted, edited, interpreted data, and prepared the manuscript. SO edited the manuscript and provided valuable suggestions during the experiment. All authors contributed to the article and approved the submitted version.

## Conflict of Interest

The authors declare that the research was conducted in the absence of any commercial or financial relationships that could be construed as a potential conflict of interest.
